# Screening for Circulating Inflammatory Proteins Does Not Reveal Plasma Biomarkers of Constant Tinnitus

**DOI:** 10.1007/s10162-023-00920-3

**Published:** 2023-12-11

**Authors:** Christopher R. Cederroth, Mun-Gwan Hong, Maxim B. Freydin, Niklas K. Edvall, Natalia Trpchevska, Carlotta Jarach, Winfried Schlee, Jochen M. Schwenk, Jose-Antonio Lopez-Escamez, Silvano Gallus, Barbara Canlon, Jan Bulla, Frances M. K. Williams

**Affiliations:** 1 Section of Experimental Audiology, Department of Physiology and Pharmacology, Karolinska Institute, Stockholm, Sweden; 2 National Institute for Health Research (NIHR) Nottingham Biomedical Research Centre, Nottingham University Hospitals NHS Trust, Ropewalk House, Nottingham, UK; 3 Department of Otolaryngology, Head and Neck Surgery, Translational Hearing Research, Tübingen Hearing Research Center, University of Tübingen, Tubingen, Germany; 4 Affinity Proteomics, Science for Life Laboratory, School of Engineering Sciences in Chemistry, Biotechnology and Health, KTH Royal Institute of Technology, Stockholm, Sweden; 5 Science for Life Laboratory, Department of Biochemistry and Biophysics, National Bioinformatics Infrastructure Sweden, Stockholm University, Stockholm, Sweden; 6 Department of Twin Research and Genetic Epidemiology, School of Life Course Sciences, King’s College London, London, UK; 7 Department of Environmental Health Sciences, Istituto di Ricerche Farmacologiche Mario Negri IRCCS, Milan, Italy; 8 Department of Psychiatry and Psychotherapy, University of Regensburg, Regensburg, Germany; 9 Faculty of Medicine & Health, School of Medical Sciences, Meniere’s Disease Neuroscience Research Program, The Kolling Institute, University of Sydney, Sydney, NSW Australia; 10 Otology and Neurotology Group CTS495, Department of Genomic Medicine, GENYO - Centre for Genomics and Oncological Research - Pfizer, University of Granada, PTS, Junta de Andalucía, Granada, Spain; 11 Division of Otolaryngology, Department of Surgery, Instituto de Investigación Biosanitaria, ibs.GRANADA, Universidad de Granada, GranadaGranada, Spain; 12 Department of Mathematics, University of Bergen, Bergen, Norway

**Keywords:** Tinnitus, Constant, Plasma, Biomarker, Diagnostic, Auditory, Olink, Profiling

## Abstract

**Background and Objective:**

Tinnitus would benefit from an objective biomarker. The goal of this study is to identify plasma biomarkers of constant and chronic tinnitus among selected circulating inflammatory proteins.

**Methods:**

A case–control retrospective study on 548 cases with constant tinnitus and 548 matched controls from the Swedish Tinnitus Outreach Project (STOP), whose plasma samples were examined using Olink’s Inflammatory panel. Replication and meta-analysis were performed using the same method on samples from the TwinsUK cohort. Participants from LifeGene, whose blood was collected in Stockholm and Umeå, were recruited to STOP for a tinnitus subtyping study. An age and sex matching was performed at the individual level. TwinsUK participants (*n* = 928) were selected based on self-reported tinnitus status over 2 to 10 years. Primary outcomes include normalized levels for 96 circulating proteins, which were used as an index test. No reference standard was available in this study.

**Results:**

After adjustment for age, sex, BMI, smoking, hearing loss, and laboratory site, the top proteins identified were FGF-21, MCP4, GDNF, CXCL9, and MCP-1; however, these were no longer statistically significant after correction for multiple testing. Stratification by sex did not yield any significant associations. Similarly, associations with hearing loss or other tinnitus-related comorbidities such as stress, anxiety, depression, hyperacusis, temporomandibular joint disorders, and headache did not yield any significant associations. Analysis in the TwinsUK failed in replicating the top candidates. Meta-analysis of STOP and TwinsUK did not reveal any significant association. Using elastic net regularization, models exhibited poor predictive capacity tinnitus based on inflammatory markers [sensitivity = 0.52 (95% CI 0.47–0.57), specificity = 0.53 (0.48–0.58), positive predictive value = 0.52 (0.47–0.56), negative predictive values = 0.53 (0.49–0.58), and AUC = 0.53 (0.49–0.56)].

**Discussion:**

Our results did not identify significant associations of the selected inflammatory proteins with constant tinnitus. Future studies examining longitudinal relations among those with more severe tinnitus and using more recent expanded proteomics platforms and sampling of cerebrospinal fluid could increase the likelihood of identifying relevant molecular biomarkers.

**Supplementary Information:**

The online version contains supplementary material available at 10.1007/s10162-023-00920-3.

## Introduction

Tinnitus is a complex neurological disorder that is characterized by the perception of phantom sounds [1]. Complexities in determining response treatment whether pharmacological [2], neuromodulatory [3], sound- [4], or psychology-based [5] result from the lack of robust biomarkers. A recent systematic review revealed conflicting evidence for the association of blood count, vitamins, lipid profile, neurotrophic factors, or inorganic ions with ill-defined tinnitus [6]. Indeed, it has been debated whether the heterogeneity of tinnitus could have been grounds to the failure in identifying biomarkers [7, 8]. Recommendations propose that larger studies, with stricter exclusion criteria and powerful harmonized methodological designs, are needed to address the current knowledge gap.

Constant tinnitus is a neurological phenomenon explained in part by failure in sensory gating mechanisms [9]. Most often it is accompanied by hearing loss or sensory deafferentation [10]. Constant tinnitus co-occur with plastic changes of the auditory pathway — once tinnitus has transitioned from being perceived occasionally to constantly it very rarely regresses and this change can also be measured as a delay of the auditory brainstem response from the inferior colliculus even when adjusted for hearing thresholds [11]. This study indeed suggests that constant tinnitus is a homogenous-enough subtype that is distinguishable by means of electrophysiology.

Tinnitus shares similar properties to chronic pain. Neuroimaging studies suggest a disturbance of the frontostriatal system, including ventromedial prefrontal cortex and the nucleus accumbens, leading to a disrupted gating mechanism for sensory input relevance and affective value [12]. Since chronic pain has recently been suggested to involve a localized inflammatory response in the brain, detectable in the blood [13, 14] or the cerebrospinal fluid (CSF) [15, 16], we hypothesized that chronic and constant tinnitus may also be associated with neuroinflammation. Indeed, recent animal studies have shown microglial activation within the auditory cortex involving TNFα [17].

Multiplexed proteomic analyses have emerged as sensitive methods to measure many potential blood biomarkers in a variety of human phenotypes [18] including cardiovascular and metabolic disease [19] and neurological disorders such as multiple sclerosis [20], Parkinson’s disease [21, 22], depression [23], and traumatic brain injury [24, 25]. We sought to identify circulating biomarkers in the plasma indicative for constant tinnitus using discovery and replication samples drawn from large studies.

## Methods

### Study Design and Ethics Statement

The present study is a case–control retrospective study to identify plasma biomarkers for constant tinnitus using a second cohort as validation, and then joining the two to perform a meta-analysis. The project has been approved by the local ethics committee “Regionala etikprövningsnämnden” in Stockholm (2015/2129–31/1). TwinsUK has ethical approval from Guys and St Thomas’ Trust Ethics Committee (REC EC04/015). Informed consent was obtained from all participants after presenting the nature and possible consequences of the studies.

### Setting and Participants

Adult participants (> 18 years old) from LifeGene [26] were recruited to the Swedish Tinnitus Outreach Project (STOP). Participants registered on the STOP website (https://stop.ki.se). After having registered, participants received detailed information and a consent form via post. Having returned the signed consent form, they were invited by secure and personal link to answer questionnaires on an online platform. Participants from TwinsUK were individuals from the UK Adult Twin Registry [27]. The TwinsUK cohort comprises healthy volunteers from the general population recruited through national media campaigns. The cohort comprises predominantly females (83%), of broad age range, mainly of Northern European descent, and includes nearly equal numbers of monozygotic and dizygotic same-sex twins. Participants have been characterized for a variety of clinical and behavioural traits longitudinally. For the purpose of the current study, participants have been selected based on the presence/absence of self-reported tinnitus, relevant covariates (age, sex, smoking, BMI, and self-reported hearing loss), and plasma availability.

### Questionnaires in STOP

**Fig. 1:**
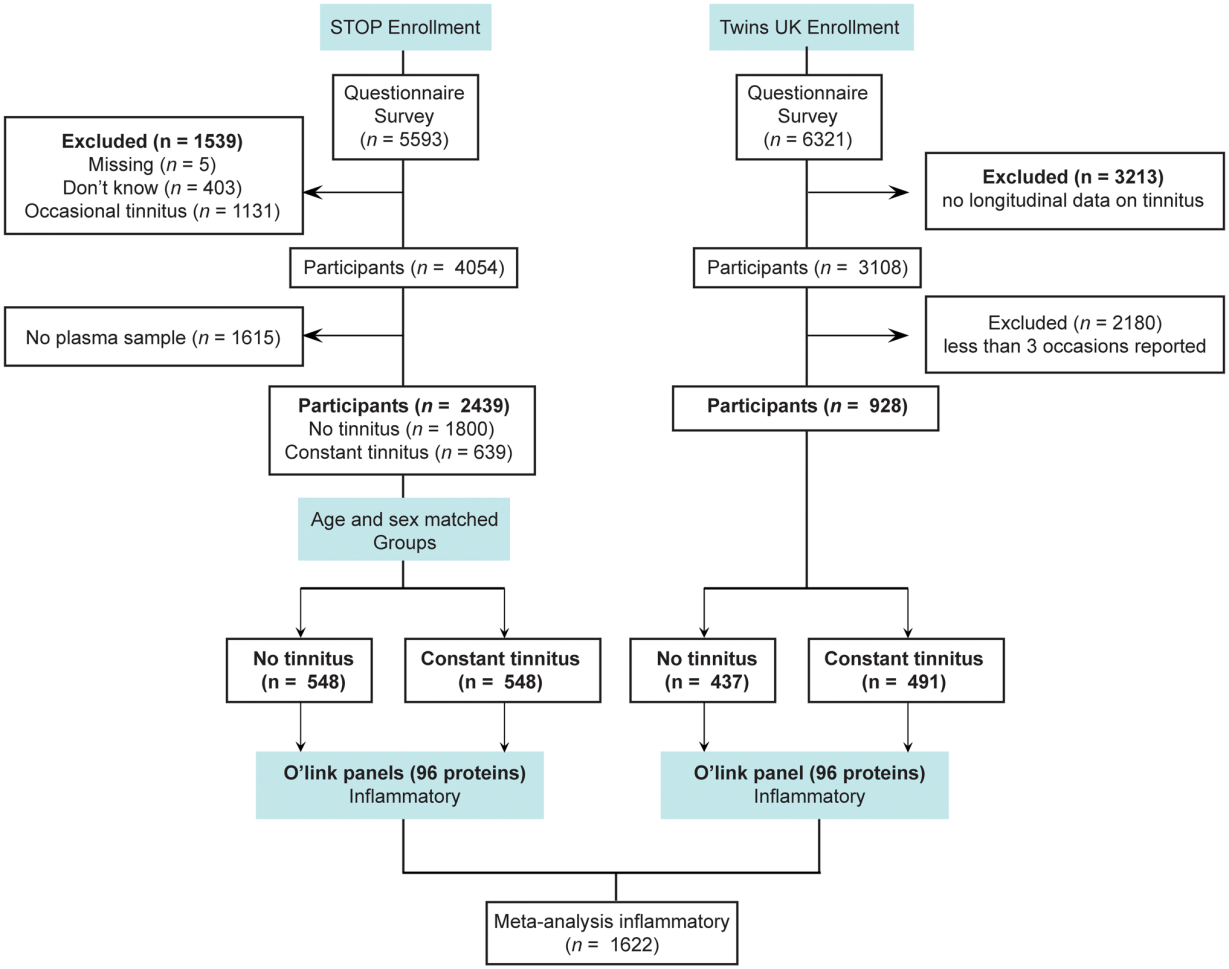
Flowchart of patient selection

Between June 2016 and January 2020, *n* = 5593 participants responded to online questionnaires. The questionnaires used were translated to Swedish, validated for online use, and have been described in detail previously [28]. In brief, the online survey consisted of the Tinnitus Sample Case History Questionnaire (TSCHQ), the Tinnitus Handicap Inventory (THI), the Tinnitus Functional Index (TFI), the Tinnitus Catastrophizing Scale (TCS), the Fear of Tinnitus Questionnaire (FTQ), the Hospital Anxiety and Depression Scale (HADS), the Perceived Stress Questionnaire (PSQ-30), the hyperacusis questionnaire (HQ), and four domains of the World Health Organisation Quality of Life Scale (WHOQoL-BREF). Two data entries on BMI were excluded possibly due to errors in data entry by the participants (eTable 1). Participants formed convenience series, whereby 1539 individuals were excluded based on an entry question “Do you have tinnitus?” (*n* = 5 missing information; *n* = 403 do not know; *n* = 1131 occasional tinnitus). As there are no established diagnostic criteria, the selection of participants remained self-reported. From the remaining 4054 participants with constant or without tinnitus, 2439 had plasma samples available (*n* = 1615 without plasma excluded), yielding 1800 participants with no tinnitus and 639 participants with constant tinnitus. Tinnitus duration was defined as “How long ago did your tinnitus start,” whereby six individuals with acute tinnitus were found (< 6 months duration). All other individuals with a tinnitus duration above 6 months were considered as chronic tinnitus. To perform a 1:1 matching with controls with same sex and age, 548 individuals were identified with a matching non-tinnitus control. This excluded 91 individuals with constant tinnitus that had no matching controls. A flowchart is presented in Fig. 1. The ESIT screening questionnaire [29] was added to the platform November 2018 and was answered by 80.9% of the full STOP participants. Six potential covariates from the ESIT-SQ, and the blood collection were tested for association with individual proteins (age, sex, BMI, smoking, sample Lab, and hearing problems). The variable code book of the two surveys used in STOP are included in the Supplemental material.

### Questionnaires in TwinsUK

Between April 2004 and December 2018, TwinsUK participants responded to self-administered questionnaires called Baseline Health Questionnaire (BHQ) and Baseline Core Questionnaire (BCQ) including the following question concerning tinnitus: “Do you suffer from tinnitus? (buzzing/ringing in the ears).” Data on hearing difficulties have been collected as responses to BHQ: “Do you suffer from hearing loss?” and in framework of the audiometry study: “Do you have any difficulty with your hearing?” [30]. Cases of tinnitus were defined as those providing a positive answer to tinnitus questions at a minimum of three time points, while controls were defined as those who repeatedly provided a negative response. Those who reported tinnitus on less than three occasions were excluded from the cases and controls resulting in *n* = 928 twins for subsequent analysis.

### Blood Sampling

For the STOP study, participants were sampled between 2011 and 2012, as well as between 2014 and 2017 at Stockholm, Stureplan, and Umeå LifeGene collection sites from 8 a. m. until 8 p. m. (eTable 2). Whole blood was collected in citrate or EDTA anticoagulant and centrifuged at 2000 g for 15 min. Plasma aliquots were snap frozen and stored at − 80 °C, and then shipped to the Karolinska Biobank. For TwinsUK participants, plasma was collected from fasting blood at the time of clinical visits.

### Blood Analysis

Analysis of 96 proteins comprising the Olink Inflammatory (v.3021 panels was performed using PEA (Proximity Extension Assay) technology. The PEA technique allows simultaneous assessment of proteins using oligonucleotide-labelled antibody probe pairs that bind to each protein within the sample [31, 32]. The PEA technique also permits accurate assessment of 92 protein levels in 1 µl of sample. The assay requires the dual recognition of a protein by matched antibody pairs, and using their DNA-barcodes, only sequence-specific oligonucleotides will be amplified to generate a data [24]. Plasma samples (25 µl) were randomly distributed to a 96 well plate (AB-0800, Thermofischer), including six Olink controls and three triplicates of a master plasma mix distributed on all 13 plates. The assay reports normalized protein expression values (NPX) as fold change in log 2 units. For this analysis, the raw data are converted into a *t*-statistic which can be compared across assays.

### Quality Control

The given data set included three negative controls (NEG) per plate, three inter-plate controls (IPC), and three to five mixed samples in addition to the clinical samples. Thirteen samples were found to produce missing values. The whole measure of one or two panels of 12 samples were missing as listed in the eTable 3. The NPX values of one sample were missing for only a couple of assays. Those missing values were imputed with medians of the assay. Some measures were below the lower limit of detection (LLOD) of the assay. The distribution of the measures of each protein below LLOD is shown in eFigure 1, where two separate clusters were observed. One of them with low proportion of LLOD indicates that the sensitivity of the assays for the proteins in the group was high enough to achieve relative quantification from the samples, whereas the assays for the other cluster could not produce comparable data. The > 50% values of 27 proteins were below LLOD, the data of which were removed. Note that the LLOD was computed per assay (or protein) for all plates. A large part (> 40%) of two sample protein values in the Inflammation panel were below LLOD. The data of the samples without protein data or with too many LLOD were removed (data not shown). A list of samples and proteins excluded is provided in eTables 4–6. The number of samples and proteins after QC were 1084 samples for 68 proteins in the inflammation panel.

### Statistical Analyses

Six potential covariates were tested for association with individual protein profiles. Several proteins were found correlated with some of those covariates (by linear regression or ANOVA, Bonferroni adjusted *p* value < 0.05). A large number of proteins were significantly associated with age, sex, BMI, hearing problems and sample lab. Those variables were included in all analyses as covariates.

The association between a protein and a clinical trait was tested using linear regression for a quantitative variable or ANOVA for a categorical variable including described covariates. Two methods were applied for multiple testing correction, Westfall and Young’s max-*T* method and *q* value. Resampling of the former method was conducted 10,000 times. The “*q* value” was computed using the *q* value (v 2.15.0) R package. Homoscedasticity assumption was checked by Bartlett’s test. Data handling and statistical analyses were conducted on R version 4.0.3 (2020–10-10), together with tidyverse (v. 1.3.1) package.

For TwinsUK, two approaches were used: total sample analysis and a discordant twin analysis. Linear mixed-effects models were fitted with proteins as dependent variables and tinnitus as independent variable adjusting for age, sex, BMI, smoking (ever vs never), and having hearing difficulties as fixed effects, and relatedness (belonging to the same family), repeated measures and twin pairing (for discordant twins analysis) as random effects. Meta-analysis between STOP and TwinsUK was carried out using fixed-effects inverse-variance weighting approach. Benjamini–Hochberg false discovery rate approach was used to adjust for multiple testing. Analyses were carried out using R packages lme4 (v 1.1.26), lmerTest (v 3.1.3), and metafor (v 2.4.0).

Elastic net regularization was used to assess diagnostic capacity of inflammatory markers for tinnitus. For this purpose, we used STOP as the train sample and TwinsUK as the test sample. Prior to elastic net regression, inflammatory markers in STOP and TwinsUK have been adjusted for sampling age, laboratory site, sex, smoking, and BMI via residuals. Best shrinkage parameter (*λ*) was chosen using tenfold cross-validation followed by fitting the elastic net regression model with mixing parameter (*α*) s set at 0.5. The model was used to assign classes of tinnitus and controls in TwinsUK setting up the probability threshold of 0.5. Contingency table for actual and predicted classes was used to estimate sensitivity, specificity, positive, and negative predictive values. Area under curve (AUC) with 95% CIs was also estimated. Analyses were carried out using R packages epiR (v 2.0.41), pROC (v 1.18.0), and glmnet (v 4.1.3).

## Results

### Sociodemographics and Characteristics of STOP Participants

A case–control approach was chosen for the discovery phase of the study. Sociodemographic information from the constant tinnitus groups and non-tinnitus controls are presented in Table 1, as well as measures of psychological and life quality impact, conventionally assessed in tinnitus studies [33]. Consistent with previous studies [34], differences between constant tinnitus and non-tinnitus controls were found for education attainment and income. Stress, anxiety, depression, and hyperacusis were more pronounced in constant tinnitus subjects. Psychological, physical, and environmental life quality were also impacted in individuals with constant tinnitus. For the constant tinnitus group, the tinnitus handicap inventory (THI) score was 20.71 (SD = 17.43) and that of the tinnitus functional index (TFI) was 22.20 (SD = 17.89) corresponding to mild tinnitus and small problem, respectively. In accordance to previous studies [35–37], the proportion of individuals with vertigo, headache, temporomandibular joint or neck pain, sensitivity to noise, and hearing difficulties was greater in the constant tinnitus group when compared to non-tinnitus controls (Table 2).

**Table 1:** Demographics and questionnaire responses from STOP participants

	**No tinnitus (*n* = 548)**	**Constant tinnitus (*n* = 548)**	***p* value**
**Sex**			1
Male	298 (54.4%)	298 (54.4%)	
Female	250 (45.6%)	250 (45.6%)	
**Age**	46.01 (12.05)	46.01 (12.05)	1
**Education**			0.009
Don’t know	1 (0.2%)	0 (0.0%)	
Middle School	8 (1.5%)	16 (3.0%)	
High School	77 (14.1%)	102 (18.8%)	
University	422 (77.0%)	368 (67.9%)	
Other	40 (7.3%)	56 (10.3%)	
**Income (× 1000 SEK/year)**			< 0.001
0–200	38 (6.9%)	52 (9.6%)	
200–450	235 (42.9%)	259 (47.8%)	
> 450	263 (48.0%)	203 (37.5%)	
Unknown	12 (2.2%)	28 (5.2%)	
**¤BMI**	24.52 (3.62)	24.91 (4.08)	0.147
**¤Smoking**			0.116
Never smoker	297 (71.7%)	275 (65.8%)	
Ex-smoker	12 (2.9%)	10 (2.4%)	
Current smoker	105 (25.4%)	133 (31.8%)	
**HADS Anxiety**	4.57 (3.46)	5.74 (4.11)	< 0.001
**HADS Depression**	2.50 (2.35)	3.37 (3.30)	< 0.001
**PSQ**	0.26 (0.16)	0.34 (0.19)	< 0.001
**HQ**	10.48 (6.67)	16.32 (8.95)	< 0.001
**QoL Physical**	17.05 (2.11)	15.97 (2.63)	< 0.001
**QoL Psychological**	16.00 (2.23)	15.15 (2.60)	< 0.001
**QoL Social**	14.65 (2.88)	14.30 (3.03)	0.05
**QoL Environment**	17.11 (1.78)	16.47 (2.25)	< 0.001
**NRS Loudness**		44.57 (24.05)	
**NRS Awareness**		37.27 (30.19)	
**NRS Annoyance**		20.77 (24.52)	
**THI**		20.71 (17.43)	
**TFI**		22.20 (17.89)	
**FTQ**		4.79 (2.43)	
**TCS**		12.84 (9.07)	

Student’s *t*-test or *χ*^2^ as appropriate. Participants in the group with no tinnitus did not answer tinnitus specific questionnaires. Categorical variables are reported as *n* (column percent); numerical variables are reported as mean (standard deviation). Items noted with ¤ are gathered from the ESIT screening questionnaire that was not answered by all participants

*BMI* body mass index, *HADS* Hospital Anxiety and Depression Scale, *PSQ* Perceived Stress Questionnaire, *HQ* Hyperacusis Questionnaire, *QoL* quality of life, *THI* tinnitus handicap inventory, *TFI* tinnitus functional index, *FTQ* fear of tinnitus questionnaire, *TCS* tinnitus catastrophizing scale

### Proteomic Profiling Reveals Associations with Age, Sex, BMI, Smoking, and Lab Sample

STOP samples were collected from three sample processing labs. The samples were balanced with respect to disease status and sex, their collection date and time (i.e., there were no bias with respect to seasonal or time-of-the-day), or plate distribution ((x)^2^ test *p* = 0.45, eFigure 2). In STOP, BMI, smoking, and hearing problem were missing for a relatively large proportion of participants (*n* = 264, 24.3%). From the resulting samples including information on BMI, smoking, and hearing problem, the average age of females was higher than males (males: 44.2 (± 0.449) and females: 48.2 (± 0.582); *t*-test, *p* = 3.67·10^−8^) (Table 2).

**Table 2:** Tinnitus related items from the ESIT-SQ for STOP participants

	**No tinnitus** ***n* = 414**	**Constant tinnitus** ***n* = 418**	***p* value**
**Vertigo**			< 0.001
Yearly or more	267 (64.5%)	191 (45.7%)	
< 1 per year	69 (16.7%)	70 (16.7%)	
Never	78 (18.8%)	157 (37.6%)	
**Headache**			< 0.001
Yes	55 (13.3%)	114 (27.3%)	
**TMJ pain**			< 0.001
Yes	14 (3.4%)	46 (11.0%)	
**Neck pain**			< 0.001
Yes	41 (9.9%)	115 (27.5%)	
**Sensitive to sounds/sound a problem?**			< 0.001
No	306 (73.9%)	164 (39.2%)	
Small	77 (18.6%)	111 (26.6%)	
Moderate	28 (6.8%)	110 (26.3%)	
Big	1 (0.2%)	29 (6.9%)	
Very big	2 (0.5%)	4 (1.0%)	
**Hearing difficulties**			< 0.001
Don’t know	4 (1.0%)	2 (0.5%)	
No difficulty	208 (50.2%)	75 (17.9%)	
Slight	134 (32.4%)	126 (30.1%)	
Moderate	56 (13.5%)	141 (33.7%)	
Severe	10 (2.4%)	71 (17.0%)	
Cannot hear	2 (0.5%)	3 (0.7%)	
**Hearing device**			< 0.001
Yes	6 (1.4%)	46 (11.0%)	

All items in Table 2 are gathered from the ESIT-SQ, that was submitted after the initial round of questionnaires and not completed by all participants. Data are represented the same way as for Table 1

A total of 96 proteins from the inflammatory panel were measured in STOP cohort. Seven potential covariates were tested for association with individual proteins (age, sex, BMI, smoking, sample Lab, collection date, and plate ID). As BMI and smoking were derived from the ESIT-SQ and only available for a subset of participants, the sample size was slightly reduced (*n* = 418 cases and *n* = 414 controls). A number of proteins were significantly associated with age, sex, BMI, and sample lab. Sixty-nine proteins were found correlated with age by linear regression. Among them, the profiles of 58 proteins (84.1%) increased and 11 decreased as age advances (eTable 7). Notably, CDCP1, CCL11, and Flt3L were the top 3 proteins increasing with age, and CD8A, NT-3, and TNFβ were the top 3 decreasing with age (eFigure 3A, B), consistent with previous studies [38, 39]. Sixty-five proteins were found correlated with sex. Among them, the profiles of 28 proteins (43.1%) were higher in females (eTable 8). The top 3 proteins in males were TRAIL, ADA, and TRANCE, while the top three proteins in females were OPG, CCL28, and CXCL5 (eFigure 3C, D). Forty-nine proteins were correlated with BMI (eTable 9). The profiles of 54 proteins (94.2%) increased with higher BMI. The top 3 were HGF, TNFSF14, and IL-18R1, as previously reported [40]. Of the six proteins that were decreased, NT-3, SCF, and CCL28 were the top three. Ten proteins were associated with smoking status (Top 3: CDCP1, IL-8, Flt3L; eFigure 3E, eTable 10). Twenty proteins were different across sample labs by ANOVA. The most highly associated three proteins were AXIN1, SIRT2, and STAMBP (eFigure 3F). Consequently, and in addition of hearing problems, age, sex, BMI, smoking, and sample lab were included as covariates in the following analyses.

#### Lack of Associations with Tinnitus or Their Associated Comorbidities

The results for all proteins that were analysed in the STOP cohort are available in Supplemental Data S1-12; herein, only the top five are reported. The top five proteins associated with tinnitus with a *p* value less than 0.02 were FGF-21, MCP4, GDNF, CXCL9, and MCP-1. However, these were no longer significant after correction for multiple testing (Table 3). In analyses stratified by sex, no significant associations were found. When testing associations between self-reported hearing loss and inflammatory proteins, no relationships were found (Table 4). As tinnitus may be accompanied by stress, anxiety, and depression [34], as well as hyperacusis [36], temporomandibular joint pain [37], and headache [35], we examined the independent associations of each of these comorbidities after adjustment for age, sex, BMI, smoking, and sample lab (Table 5; Supplemental Data S7-12). There too, no significant associations were found.

**Table 3:** Top proteins in relation to constant tinnitus in STOP participants

**Protein**	**∆(Yes–No)**	***Std. error***	***p*** ***value***	***q*** ***value***	***Perm.*** ***value***
*Both sexes*					
FGF-21	0.290	0.095	0.002	0.165	0.121
MCP-4	0.143	0.050	0.005	0.304	0.206
GDNF	0.072	0.028	0.011	0.740	0.410
CXCL9	0.155	0.064	0.016	1	0.526
MCP-1	0.079	0.034	0.020	1	0.598
*Males only*					
TGF-β1	0.116	0.045	0.010	0.706	0.394
MCP-4	0.171	0.067	0.011	0.712	0.394
MMP-1	0.292	0.114	0.011	0.725	0.394
MCP-1	0.124	0.048	0.011	0.744	0.396
GDNF	0.089	0.039	0.024	1	0.654
*Females only*					
FGF-21	0.378	0.133	0.005	0.327	0.230
SCF	0.101	0.044	0.022	1	0.651
FGF-19	− 0.242	0.107	0.025	1	0.686
PD-L1	− 0.116	0.054	0.034	1	0.786
CSF-1	− 0.041	0.025	0.104	1	0.992

The effect size, labelled as ∆ ‘(Yes–No)’ in the following tables, is the difference between tinnitus cases and controls of estimated protein values after adjustment for covariates (age, sex, BMI, sample lab, smoking, and hearing problem). Positive value indicates the estimated value was higher in cases than controls. Please note that absolute magnitudes are not comparable between proteins, because the NPX values from Olink assays are given in arbitrary units

**Table 4:** Top proteins in relation to self-reported hearing problems in STOP participants

**Protein**	**∆(Yes—No)**	***Std. Error***	***p*** ***value***	***q*** ***value***	***Perm.*** ***value***
*Both sexes*					
IL-6	− 0.138	0.050	0.006	0.401	0.262
AXIN1	− 0.173	0.071	0.016	1.000	0.533
TNFSF14	− 0.071	0.033	0.029	1.000	0.737
CD40	− 0.049	0.023	0.034	1.000	0.794
CXCL11	− 0.113	0.059	0.054	1.000	0.917
*Males only*					
SCF	0.065	0.032	0.047	1.000	0.879
DNER	0.049	0.025	0.051	1.000	0.894
uPA	0.052	0.029	0.076	1.000	0.966
CX3CL1	0.067	0.039	0.088	1.000	0.980
IL-7	− 0.109	0.064	0.088	1.000	0.980
*Females only*					
CD40	− 0.106	0.035	0.003	0.191	0.140
VEGFA	− 0.096	0.032	0.003	0.205	0.147
TGF-α	− 0.073	0.026	0.006	0.385	0.254
TNFSF14	− 0.126	0.050	0.012	0.784	0.431
IL-6	− 0.199	0.081	0.015	1.000	0.504

The effect size, labelled as ∆ ‘(Yes–No)’ in the following tables, is the difference between individuals with or without self-reported hearing problems of estimated protein values after adjustment for covariates (age, sex, BMI, sample lab, smoking). Positive value indicates the estimated value was higher in individuals with hearing loss than those without. Please note that absolute magnitudes are not comparable between proteins, because the NPX values from Olink assays are given in arbitrary units

#### Replication in TwinsUK and Meta-analysis

To verify whether the top candidate proteins found in the STOP cohort (Table 3) could play a role in constant tinnitus, we sought to replicate these findings using the TwinsUK cohort. The sample comprised *n* = 928 twins, of which 433 have been analysed with Olink Inflammation panel repeatedly (correspondingly, the total sample size was *n* = 1361) (Table 6). The sample included 491 cases of tinnitus and 437 controls. There were 92 males and 836 females. Mean age was 57.0 ± 10.4 years; mean BMI was 26.1 ± 4.6 kg/m^2^. There were 287 complete pairs of twins, including 172 pairs discordant for tinnitus.

**Table 5:** Top proteins in relation to various tinnitus co-morbidities in STOP participants

**Protein**	**∆(Yes–No)**	***Std. error***	***p*** ***value***	***q*** ***value***	***Perm.*** ***value***
*Stress (PSQ-30)*					
MMP-10	0.319	0.120	0.008	0.549	0.338
SCF	0.142	0.068	0.038	1	0.829
CXCL10	− 0.326	0.175	0.062	1	0.947
TNF	− 0.223	0.138	0.107	1	0.993
IL-10	− 0.182	0.120	0.132	1	0.998
*Anxiety (HADS_a)*					
ADA	− 0.006	0.003	0.070	1	0.965
CD8A	− 0.010	0.006	0.108	1	0.994
DNER	− 0.003	0.002	0.110	1	0.995
CXCL11	− 0.011	0.007	0.129	1	0.997
GDNF	− 0.005	0.003	0.146	1	0.999
*Depression (HADS_d)*					
MMP-10	0.018	0.007	0.010	0.655	0.390
IL-6	0.014	0.008	0.069	1	0.957
CCL25	− 0.011	0.006	0.075	1	0.966
TGF-α	0.006	0.004	0.083	1	0.975
CD8A	− 0.013	0.008	0.094	1	0.984
*Hyperacusis (HQ)*					
SCF	0.004	0.002	0.006	0.396	0.267
LIF-R	0.002	0.001	0.061	1	0.945
CXCL5	0.08	0.005	0.078	1	0.977
CXCL10	− 0.006	0.004	0.089	1	0.986
SIRT2	− 0.006	0.004	0.117	1	0.995
*TMJ pain (ESTI-SQ A15_4)*					
ADA	0.104	0.031	0.001	0.063	0.055
MCP-1	0.093	0.035	0.008	0.553	0.345
IL-8	0.108	0.047	0.021	1	0.635
CCL28	0.078	0.034	0.023	1	0.656
CXCL11	0.142	0.064	0.028	1	0.727
*Headache (ESTI-SQ A15_1)*					
GDNF	0.056	0.021	0.006	0.402	0.263
CXCL9	0.127	0.047	0.007	0.466	0.289
FGF-21	0.199	0.074	0.008	0.513	0.308
MMP-1	0.167	0.064	0.009	0.600	0.345
MCP-1	0.062	0.024	0.011	0.725	0.390

The effect size, labelled as ∆ ‘(Yes–No)’ in the following tables, is the difference between individuals with or without self-reported hearing problems of estimated protein values after adjustment for covariates (age, sex, BMI, sample lab, smoking). Positive value indicates the estimated value was higher in individuals with the given comorbidity than those without. Please note that absolute magnitudes are not comparable between proteins, because the NPX values from Olink assays are given in arbitrary units

**Table 6:** Demographics of twins from TwinsUK between April 2004 and December 2018

	**No tinnitus (n = 491)**	**Constant tinnitus (n = 437)**	**p value**
**Age, years**	56.3 (11.2)	57.8 (10.1)	0.0328
**Sex**			0.025
Male	38 (7.7%)	54 (12.4%)	
Female	453 (92.2%)	383 (87.6%)	
**BMI, kg/m**^**2**^	26.0 (4.5)	26.3 (4.7)	0.369
**Hearing loss**			2.2e − 16
Yes	169 (34.4%)	284 (65%)	
No	322 (65.6%)	153 (35%)	
**Smoking**			0.456
Ever	224 (45.6%)	211 (48.3%)	
Never	267 (54.4%)	226 (51.7%)	

Student’s *t*-test or *χ*^2^ as appropriate

*BMI* body mass index

The total of 92 proteins were measured in TwinsUK, of which we retained those with the number of samples having results below LOD less than 50% and those that were analysed in STOP regardless of the number of samples below LOD to allow meta-analysis. This resulted in 73 proteins for examination.

After correction for multiple testing, no statistically significant results were achieved (Supplemental Data S13, S14). Proteins showing significance before correction for association with tinnitus were NT-3, uPA, and CX3CL1 when using the whole sample, and NT-3, CX3CL1, and CCL3 when using discordant twins (Table 7).

**Table 7:** Top proteins in relation to constant tinnitus in TwinsUK

**Protein**	**Estimate**	***Std.*** ***Error***	***p*** ***value***	***t*** ***value***	**FDR**
*Total sample*					
NT.3	0.071	0.025	0.004	2.887	0.292
uPA	0.040	0.020	0.039	2.071	0.845
CX3CL1	0.049	0.024	0.043	2.029	0.845
TWEAK	0.039	0.021	0.063	1.862	0.845
SCF	0.041	0.022	0.066	1.839	0.845
*Discordant twins*					
NT.3	0.111	0.036	0.002	3.140	3.140
CX3CL1	0.087	0.031	0.005	2.815	2.820
CCL3	− 0.101	0.043	0.019	− 2.350	− 2.350
PD.L1	0.057	0.030	0.056	1.923	1.923
Flt3L	0.053	0.028	0.056	1.916	1.916

Linear mixed-effects models were fitted with proteins as dependent variables and tinnitus as independent variable adjusting for age, sex, BMI, smoking (ever vs never), and having hearing difficulties as fixed effects, and relatedness (belonging to the same family), repeated measures and twin pairing (for discordant twins analysis) as random effects. Total samples and discordant twins only models were considered. Top 5 proteins are shown; full results are presented in Tables S13 and S14

None of the top 5 candidate proteins detected in STOP cohort (Table 3) was replicated in TwinsUK by either method. Meta-analysis of STOP and TwinsUK datasets did not reveal any statistically significant associations after correction for multiple testing (Supplemental Data S15, S16). Results significant before correction were obtained for TWEAK, MCP-1, CX3CL1, SCF, MCP-2, MCP-4, and CCL25 proteins when using the whole TwinsUK sample, and CX3CL1, GDNF, Flt3, MCP-2, TWEAK, MCP-4, CCL11, and CXCL1 when using discordant twin pairs (Table 8). The majority of twins were females, thus we repeated the meta-analysis restricted to females only in the STOP and TwinsUK cohorts. Before correction, significant constant tinnitus was associated with SCF, TWEAK, and CX3CL1 using the whole TwinsUK sample, and CX3CL1 using the discordant twin pairs (Supplemental Data S17, S18).

**Table 8:** Meta-analysis between STOP and TwinsUK

**Protein**	**Effect**	***Std. Error***	**95% CI**	***Z***	***p* value**	***I***^**2**^	***Q***	***Qp***	**LOD**	**FDR**
*Total TwinsUK sample*										
TWEAK	0.037	0.016	0.006; 0.048	2.35	0.019	0	0.014	0.907	0%	0,422
MCP-1	0.047	0.021	0.006; 0.058	2.219	0.026	31.8	1.466	0.226	0%	0,422
CX3CL1	0.042	0.019	0.005; 0.051	2.197	0.028	0	0.218	0.64	0%	0,422
SCF	0.038	0.017	0.005; 0.047	2.184	0.029	0	0.044	0.834	0%	0,422
MCP-2	0.063	0.029	0.006; 0.075	2.157	0.031	0	0.001	0.97	0%	0,422
MCP-4	0.061	0.031	2e − 4; 0.061	1.996	0.046	76.5	4.248	0.039	0%	0,466
CCL25	0.048	0.024	0.001; 0.050	1.979	0.048	0	0.604	0.437	0%	0,466
*Discordant twins only*										
CX3CL1	0.059	0.022	0.016; 0.090	2.684	0.007	39.4	1.65	0.199	0%	0,359
GDNF	0.048	0.02	0.009; 0.065	2.344	0.019	35.4	1.547	0.214	68%	0,359
Flt3L	0.044	0.021	0.003; 0.050	2.157	0.031	0	0.212	0.645	0%	0,359
MCP-2	0.071	0.033	0.006; 0.083	2.146	0.032	0	0.045	0.833	0%	0,359
TWEAK	0.039	0.018	0.004; 0.046	2.15	0.032	0	0.079	0.779	0%	0,359
MCP-4	0.072	0.034	0.005; 0.083	2.134	0.033	72.7	3.666	0.056	0%	0,359
CCL11	0. 051	0.024	0.004; 0.059	2.09	0.037	0	0.405	0.525	0%	0,359
CXCL1	0.059	0.022	0.016; 0.090	2.684	0.007	39.4	1.65	0.199	0%	0,359

Fixed-effects meta-analysis was carried out between STOP and TwinsUK cohorts. Nominally significant results (significant before correction, *p* < 0.05) are presented; full results are provided in Table S15 and S16. *I*^2^ and *Q*, heterogeneity statistics; *Qp*, *p*-value for *Q*; LOD, percentage of individuals that did not pass limit of detection in Olink assay; FDR, false-discovery rate adjusted

#### Poor Prediction of Constant Tinnitus Using Inflammatory Biomarkers

Using elastic net regularization, we developed a predictive model for tinnitus based on inflammatory markers (Table 9). The model exhibited poor predictive capacity: sensitivity = 0.52 (95% CI 0.47–0.57), specificity = 0.53 (0.48–0.58), positive predictive value = 0.52 (0.47–0.56), negative predictive values = 0.53 (0.49–0.58), and AUC = 0.53 (0.49–0.56).

**Table 9:** Predictive model for tinnitus

**Parameter**	**Coefficient**
(Intercept)	0.002
GDNF	0.244
IL-7	0.031
CXCL9	0.073
CXCL1	− 0.027
TGF-alpha	0.027
MCP-4	0.133
MMP-1	0.020
FGF-21	0.095
PD-L1	− 0.230
CXCL5	− 0.033
IL-12B	0.003
MMP-10	− 0.011
EN-RAGE	− 0.020
NT-3	− 0.119
TWEAK	0.002

Elastic net regularization was used to assess diagnostic capacity of inflammatory markers for tinnitus. The model is based on STOP cohort as the train sample. Prior to elastic net regression, inflammatory markers in STOP have been adjusted for sampling age, laboratory site, sex, smoking, and BMI via residuals. Best shrinkage parameter (*λ*) was chosen using tenfold cross-validation followed by fitting the elastic net regression model with mixing parameter (*α*) set at 0.5

## Discussion

The present study strongly supports the lack of association between plasma inflammatory biomarkers and constant tinnitus in the European population. We used two large cohorts of subjects with constant tinnitus in Sweden (548 cases and 548 controls) and in the UK (491 cases and 437 controls), the combination of which was leveraged to perform a meta-analysis. Thus, our findings are going against the notion that protein biomarkers for tinnitus may be found in the blood. This contrasts our recent report revealing an increased latency of the Wave V of the auditory brainstem response from individuals with constant tinnitus, when compared to those with occasional tinnitus or non-tinnitus controls [11]. The present analyses were carried out adjusting for factors that have a large impact on the inflammatory makers and tinnitus such as age, sex, BMI, and smoking, and hearing loss. These are the conclusions from two large studies, one that has been performed from fasting samples (TwinsUK) and the other from non-fasting samples (STOP), but where there were no differences in the collection date or time of the day. While the internal validity of these two studies is strong, the meta-analysis may have been impacted by the difference in fasting state between STOP and the TwinsUK. Also, the results of meta-analysis might have potentially been affected by the use of a non-twin sample and twins. However, this is unlikely given that we used the adjustment for kinship in twins and also the fact that twins from TwinsUK are representative of the general population and have been used in meta-analytical omics studies for decades without any noticeable impact of their relatedness [27]. Overall, it appears that while biomarkers can be derived from electrophysiological measures, this does not appear to be the case for blood inflammatory biomarkers, even with a careful control over confounding factors.

The aetiology of tinnitus (e.g., noise-exposed, objective, subjective) as well as other definitions of tinnitus may have been insufficiently precise to obtain an homogeneous enough group. History on noise exposure could not be obtained from the current datasets, nor were we able to infer occupational noise exposure from work-related activities. In contrast, 235 out of 550 STOP participants with tinnitus reported blast-noise exposure. However since this information was collected with the TSCHQ questionnaire during the establishment of the cohort, it was only submitted to participants with tinnitus, not the controls. Such information will be important to collect in future studies not only in individuals with tinnitus but also in controls. Recent epidemiological studies that defined specific subgroups of tinnitus have been successful in revealing a high heritability for bilateral tinnitus in twins [41], clinically significant tinnitus in adoptees [42], and a strong familial aggregation for severe tinnitus [43], highlighting the relevance of tinnitus definitions to examine a homogeneous subgroup, at least from a genetics perspective. Consistently, a whole exome study of tinnitus patients with an extreme phenotype has identified a set of replicable rare missense variants [44]. Nonetheless, extreme phenotypes in tinnitus are very rare (< 1% of the population), and should such phenotypes be more amenable to biomarker discovery, then a greater biobanking effort will be needed to gather such patients, not only for genetic studies but also for blood analyses [45].

As an aging phenotype, tinnitus is likely to be confounded by other common conditions of aging, related and unrelated to the tinnitus, such as cardiovascular disease and osteoarthritis. Thus, if inflammation is truly a mechanism of importance in the inner ear or in the brain, it is likely very localized and not amenable to assay on blood testing. To our knowledge, protein measures in the cochlea, in the brain, or the CSF of tinnitus subjects have not been performed as yet, but such studies may substantially increase the knowledge on the pathophysiology of tinnitus. For instance, an increasing number of studies involve multi-omic investigations to assess the genetic effects on proteins in specific traits [46]. The lack of ear-specific human tissue with either mRNA or protein expression is a major limitation that needs to be addressed, but the recent proteome of the human brain and the CSF [47] may prove more valuable in the context of tinnitus. While CSF may provide further useful information closer to the site of pathology in tinnitus, it seems unlikely that routine CSF collection will ever contribute to the clinical management of tinnitus. Imaging or electrophysiological biomarkers, however, may be more relevant. Several studies point towards the involvement of limbic structures in individuals with tinnitus [48, 49]. Likewise, other studies evaluating tinnitus by means of electrophysiology have revealed that tinnitus is related to an increased latency of the Wave V of the auditory brainstem response [11, 50]. Importantly, these studies either stratify by hearing loss or hyperacusis, or adjust their analysis taking major confounders into account. We acknowledge that recent reports point at an influencing role of medication on blood protein levels. This type of information could not be retrieved from our participant. It thus remains unclear how medication may have masked the potential association of some biomarkers with tinnitus.

It may be argued that our sample size may not have sufficed to reveal positive associations. Using Cohen’s procedure, we estimated that with the STOP sample alone (*n* = 694) we had 80% power to detect an effect size of *f* = 0.11 for a single ANCOVA test, the value just above small effect according to Cohen’s benchmarking (*f* = 0.10, small effect; *f* = 0.25, medium effect; *f* = 0.40, large effect). A combined STOP and TwinsUK sample (*n* = 1622) achieved 80% power for *f* = 0.07. Taking into account multiple testing (with 68 proteins in the meta-analysis, *α* = 0.05/68 = 0.0007), 80% power is achieved for *f* = 0.11 for meta-analysis. Thus, we may expect to detect small effects with meta-analysis even for the large number of proteins. A potential limitation of our work is our inability to replicate data reported in few studies. Indeed, a relationship between interleukin levels and tinnitus has been suggested. For instance, IL-1β was found in 30 patients with chronic tinnitus to correlate with distress levels, as well as tinnitus awareness [51], and IL-10 was found lower in subjects with tinnitus when compared to those without tinnitus (*n* = 114) [52]. Likewise, neurotrophic factors have been for long been hypothesized as contributors to tinnitus. Conflicting studies revealed either lower or higher levels of plasma BDNF in baseline individuals with tinnitus when compared to non-tinnitus controls [53, 54]. While our panels did not include proteins such as IL-1β or BDNF, our analysis suggest that IL-10 plays no role in constant tinnitus. With regard to BDNF, its measure in plasma is less convenient than in serum, with concentrations being near a 100-fold lower in the plasma [55], and being affected by handling of the blood sample (e.g., shearing forces during blood withdrawal) [56]. Thus, the quantification of BDNF is very difficult to achieve from plasma samples. New technologies may enable to re-evaluate the possible link between BDNF and tinnitus. Furthermore, the present study only examined one Olink proteomics panel. Newer Olink platforms assessing more than 3000 proteins may increase the chances of discovering biomarkers for tinnitus.

Another limitation is the fact that blood was not collected at the same time as tinnitus was evaluated in STOP. Indeed, STOP is a collaboration with LifeGene, who collected blood from 2011 to 2017. This large time span may also have influenced the outcome. However, 18.26% of the STOP participants reported having tinnitus < 5 years when participating in the survey between June 2016 and January 2020. We thus believe that this discrepancy may have a negligible impact on the present results. We recommend in future biobanking efforts that blood is collected from cases and controls at the same time as data on tinnitus is obtained [45].

### Class of Evidence

This is a diagnostic accuracy study with a case–control study design. A large number of tinnitus cases and controls were matched according to specific eligibility criteria. All cases and controls were objectively compared for possible associations with selected biomarkers. The results of biomarkers were determined without knowing the tinnitus status. For all these reasons, the present study is classified as a Class II study.

## Conclusion

In a screen for a subset of 96 inflammatory proteins from the Olink system, our large study of constant tinnitus in two cohorts did not reveal evidence of systemic inflammatory processes related to tinnitus. Future endeavours focusing on more severe tinnitus phenotypes and sampling of cerebrospinal fluid using more recent expanded proteomics platforms could increase the likelihood of identifying relevant molecular biomarkers.

### Supplementary Information

Below is the link to the electronic supplementary material.

Supplementary file1 (XLSX 165 KB)

Supplementary file2 (DOCX 4145 KB)

## Data Availability

The anonymized patient data are not being publicly shared as they are being utilized for the development of multiomic analyses within the context of the UNITI trial. All data and related documentation underlying the reported results will be made available after anonymization of patient information. Data will be made available after publication of this article: the authors will share the data with qualified investigators whose proposal of data use has been approved by an independent review committee. Full code is available in the following GitHub repositories (https://github.com/translational-audiology-lab/STOP_bloodscreen; https://github.com/translational-audiology-lab/UKTWINS_bloodscreen).
