# Supplementary material for: Screening for Circulating Inflammatory Proteins Does Not Reveal Plasma Biomarkers of Constant Tinnitus
Source: J Assoc Res Otolaryngol. 2023 Dec 11;24(6):593–606. doi: 10.1007/s10162-023-00920-3 (PMC10752855; doi:10.1007/s10162-023-00920-3)
Supplement: Supplementary file 2 — Supplementary file2 (DOCX 4145 KB) [file 10162_2023_920_MOESM2_ESM.docx]

Supplementary Materials:


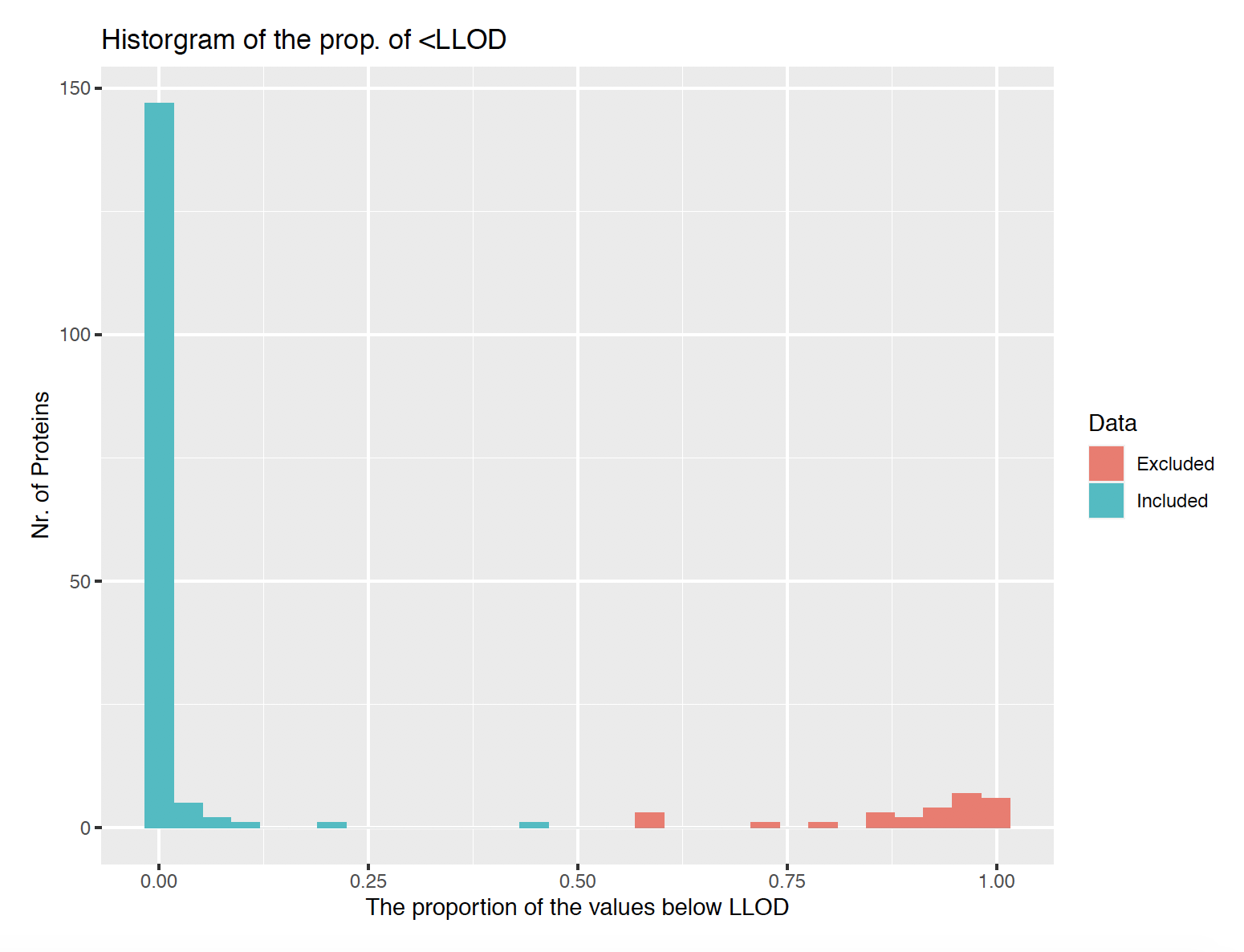


**eFigure 1.** Exclusion of STOP samples below LOD


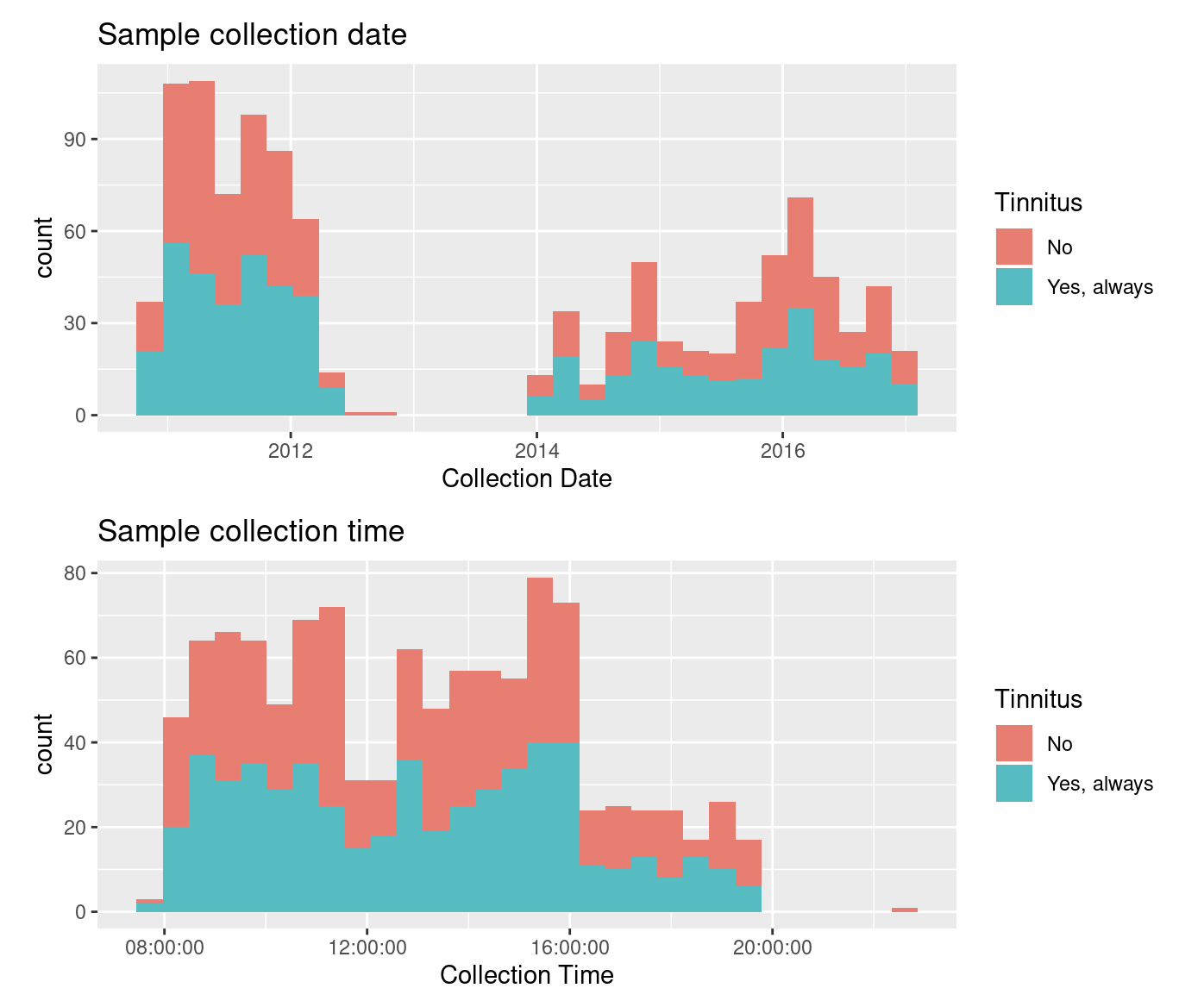


**eFigure 2**. **Distribution of the STOP samples according to date of collection or collection time during the day.** No tinnitus controls are shown in red, and constant tinnitus is shown in blue.


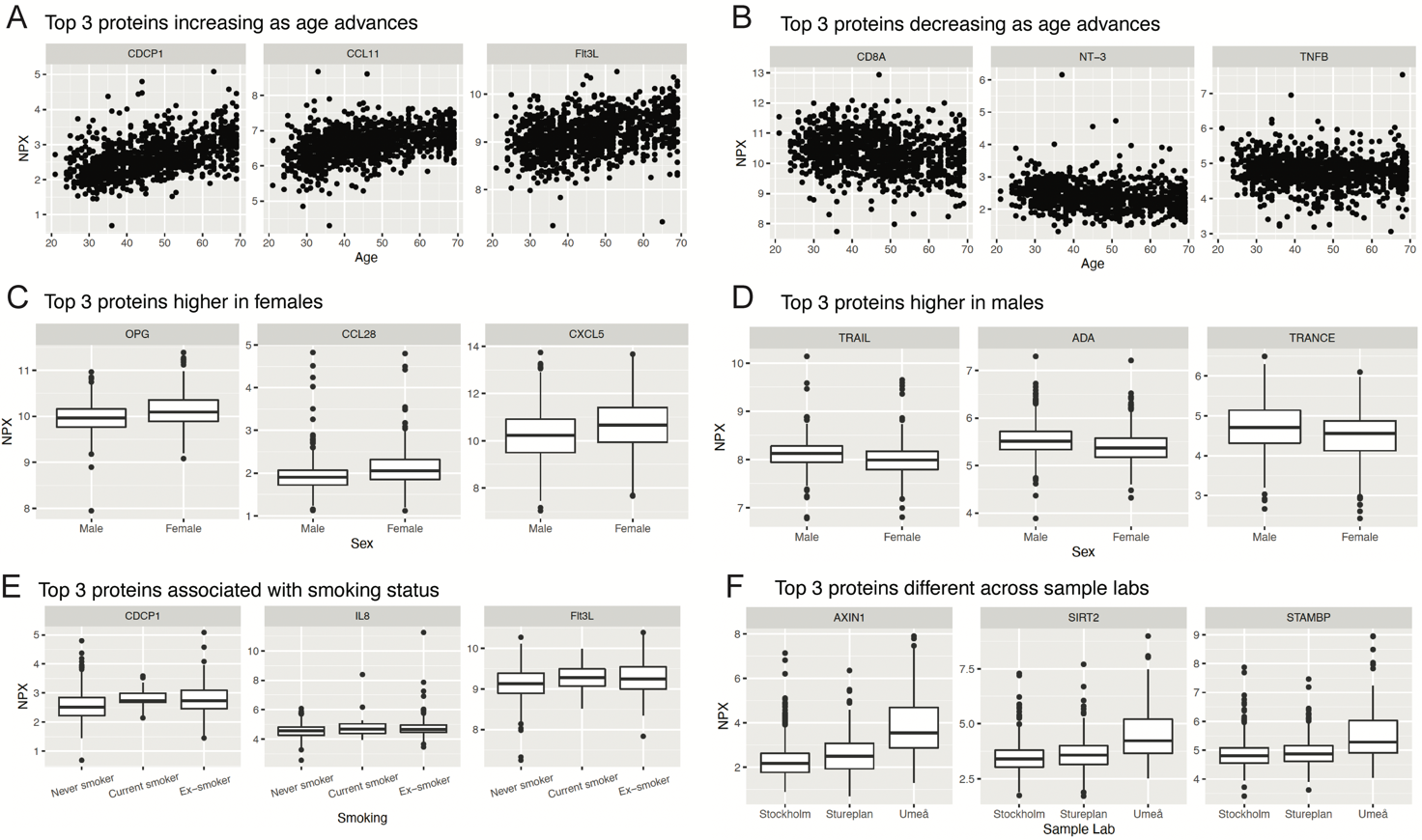


**eFigure 3**. **Association of plasma protein in STOP with selected variables.** Top 3 proteins in relation to increasing (A) or decreasing (B) age, female (C) or male (D) sex, smoking status (E) or sample lab (F).

| RID | Age | Sex | BMI | Height (cm) | Weight (kg) |
| --- | --- | --- | --- | --- | --- |
| 4993 | 68 | Male | 1.5 | 180 | 5 |
| 92855 | 53 | Female | 103.6 | 78 | 63 |

**eTable 1.** Exclusion of BMI samples. BMI was computed using height and weight. Two extreme values were observed. Because they seem to be typos / erroneous, these two samples were excluded manually.

| Sample Lab | Tinnitus | Male | Female | P-value* | Avg. Sampling Age |
| --- | --- | --- | --- | --- | --- |
| Stockholm | No | 139 | 99 | 0.102 | 41.2 |
| Stockholm | Yes, always | 127 | 123 | 0.102 | 41.2 |
| Umeå | No | 28 | 28 | 0.257 | 34.7 |
| Umeå | Yes, always | 36 | 22 | 0.257 | 34.7 |
| Stureplan | No | 131 | 123 | 0.321 | 44.5 |
| Stureplan | Yes, always | 135 | 105 | 0.321 | 44.5 |

**eTable 2.** Age and sex of the samples from each sample lab.

| Sample ID | Plate ID | Inflammation |
| --- | --- | --- |
| 18371 | 1 | Missing |
| 47636 | 1 | Missing |
| 16679 | 3 | Missing |
| 11619 | 6 | Missing |
| 13847 | 6 | Missing |
| 4830 | 6 | Missing |
| 9484 | 7 | Missing |

**eTable 3.** Missing values in thirteen samples.

| RID | Tinnitus | Sampling Age | Sample Lab | |
| --- | --- | --- | --- | --- |
| Inflammation | | | |  |
| 18371 | Yes, always | 26 | Stockholm | |
| 47636 | Yes, always | 33 | Stockholm | |
| 16679 | No | 35 | Stockholm | |
| 4830 | Yes, always | 42 | Umeå | |
| 13847 | No | 27 | Umeå | |
| 11519 | Yes, always | 40 | Stockholm | |
| 9484 | No | 45 | Stockholm | |

**eTable 4.** Samples without any protein values.

| RID | Tinnitus | Sampling Age | Sample Lab | Panel | LLOD prop. |
| --- | --- | --- | --- | --- | --- |
| 37290 | Yes, always | 49 | Umeå | Inflammation | 45% |
| 7114 | No | 21 | Stockholm | Inflammation | 99% |

**eTable 5.** Samples having too many values below LLOD.

| OlinkID | | UniProt ID | Protein | LOD | LLOD prop. | |
| --- | --- | --- | --- | --- | --- | --- |
| Inflammation | | | | |  |  |
| OID00495 | | P60568 | IL2 | 1.73154 | 100% | |
| OID00497 | | Q969D9 | TSLP | 1.48141 | 99% | |
| OID00502 | | Q13291 | SLAMF1 | 2.53960 | 99% | |
| OID00483 | | Q9P0M4 | IL-17C | 2.24645 | 98% | |
| OID00493 | | P01583 | IL-1 alpha | -0.12925 | 98% | |
| OID00516 | | Q8N6P7 | IL-22 RA1 | 3.67836 | 98% | |
| OID00543 | | O95760 | IL33 | 1.53637 | 98% | |
| OID00492 | | P14784 | IL-2RB | 2.39212 | 97% | |
| OID00519 | | P01138 | Beta-NGF | 1.37343 | 97% | |
| OID00524 | | Q13007 | IL-24 | 2.56993 | 97% | |
| OID00537 | | Q9NYY1 | IL-20 | 0.91884 | 96% | |
| OID00547 | | P15018 | LIF | 0.96513 | 94% | |
| OID00548 | | Q99748 | NRTN | 1.22507 | 94% | |
| OID00489 | | Q9UHF4 | IL-20RA | 1.61594 | 92% | |
| OID00526 | | Q5T4W7 | ARTN | 1.09351 | 92% | |
| OID00525 | | P35225 | IL13 | 1.75993 | 90% | |
| OID00546 | | P05112 | IL4 | 1.37934 | 89% | |
| OID00559 | | P05113 | IL5 | 1.71393 | 87% | |
| OID00474 | | P80098 | MCP-3 | 2.13576 | 86% | |
| OID00509 | | P12034 | FGF-5 | 1.07298 | 86% | |
| OID00485 | | Q16552 | IL-17A | 1.70762 | 80% | |
| OID00550 | | Q14790 | CASP-8 | 1.33608 | 71% | |
| OID00508 | | Q13651 | IL-10RA | 1.00553 | 60% | |
| OID00557 | | P50225 | ST1A1 | 1.81126 | 57% | |

**eTable 6.** Assays having too many values below LLOD.

| Protein | OlinkID | Estimate | Std. Error | t value | Pval | P_bonf* |
| --- | --- | --- | --- | --- | --- | --- |
| ***Increasing proteins as age advances*** | | | | | | |
| CDCP1 | OID00476 | 0,02172486 | 0,00111068 | 19,5599202 | 3,464E-73 | 2,3555E-71 |
| CCL11 | OID00505 | 0,01368609 | 0,00102786 | 13,3150972 | 1,4342E-37 | 9,7526E-36 |
| Flt3L | OID00533 | 0,01185149 | 0,00094627 | 12,5243631 | 1,0538E-33 | 7,1656E-32 |
| MCP-4 | OID00504 | 0,01642906 | 0,00155062 | 10,5951482 | 5,028E-25 | 3,4191E-23 |
| CXCL9 | OID00490 | 0,01954861 | 0,00185978 | 10,5112496 | 1,1301E-24 | 7,6845E-23 |
| MCP-1 | OID00484 | 0,00966747 | 0,00098047 | 9,8600283 | 5,0863E-22 | 3,4587E-20 |
| CCL25 | OID00551 | 0,01192464 | 0,0012457 | 9,57262587 | 6,8138E-21 | 4,6334E-19 |
| HGF | OID00522 | 0,00721008 | 0,00084043 | 8,57908394 | 3,2728E-17 | 2,2255E-15 |
| FGF-21 | OID00512 | 0,02538451 | 0,00297156 | 8,54248859 | 4,4059E-17 | 2,996E-15 |
| OPG | OID00479 | 0,00701867 | 0,00082366 | 8,52129647 | 5,2311E-17 | 3,5572E-15 |
| IL8 | OID00471 | 0,0109497 | 0,00129723 | 8,44084765 | 1,0005E-16 | 6,8031E-15 |
| CCL3 | OID00532 | 0,01028689 | 0,00126625 | 8,12390997 | 1,2229E-15 | 8,316E-14 |
| IL6 | OID00482 | 0,01218233 | 0,00161124 | 7,56083359 | 8,5212E-14 | 5,7944E-12 |
| VEGFA | OID00472 | 0,00536414 | 0,000711 | 7,5444679 | 9,6019E-14 | 6,5293E-12 |
| CST5 | OID00491 | 0,00819236 | 0,00118614 | 6,90674372 | 8,4436E-12 | 5,7417E-10 |
| CXCL10 | OID00535 | 0,01342518 | 0,00212873 | 6,30667124 | 4,1468E-10 | 2,8198E-08 |
| CD40 | OID00542 | 0,00482645 | 0,0007687 | 6,2787 | 4,9344E-10 | 3,3554E-08 |
| IL-15RA | OID00514 | 0,00404308 | 0,00065046 | 6,21568661 | 7,2823E-10 | 4,952E-08 |
| CCL28 | OID00539 | 0,00585477 | 0,00096114 | 6,09146035 | 1,5527E-09 | 1,0558E-07 |
| MMP-1 | OID00510 | 0,01365753 | 0,00255207 | 5,35155757 | 1,0641E-07 | 7,236E-06 |
| IL18 | OID00501 | 0,00642886 | 0,00129066 | 4,98106566 | 7,3561E-07 | 5,0022E-05 |
| CCL4 | OID00498 | 0,00698406 | 0,00143009 | 4,88366144 | 1,1981E-06 | 8,1473E-05 |
| MCP-2 | OID00549 | 0,00653317 | 0,00145779 | 4,48157475 | 8,1977E-06 | 0,00055744 |
| FGF-19 | OID00545 | 0,00998462 | 0,00227859 | 4,38193139 | 1,2907E-05 | 0,0008777 |
| CXCL5 | OID00520 | 0,01123366 | 0,00275036 | 4,08442836 | 4,7444E-05 | 0,00322617 |
| IFN-γ | OID05547 | 0,0097713 | 0,00241781 | 4,04138881 | 5,6895E-05 | 0,00386886 |
| LAP TGF-β1 | OID00480 | 0,00391807 | 0,00102699 | 3,81512084 | 0,00014382 | 0,00977972 |
| CXCL11 | OID00486 | 0,00678148 | 0,00183036 | 3,70498856 | 0,00022209 | 0,01510216 |
| CXCL1 | OID00496 | 0,00731079 | 0,00208068 | 3,51366258 | 0,00046024 | 0,03129599 |
| TRAIL | OID00488 | 0,0028166 | 0,00080269 | 3,50896145 | 0,00046835 | 0,03184802 |
| IL-18R1 | OID00517 | 0,00331156 | 0,00099934 | 3,31374628 | 0,00095105 | 0,0646712 |
| uPA | OID00481 | 0,00225443 | 0,00074707 | 3,01769928 | 0,00260661 | 0,17724917 |
| FGF-23 | OID00507 | 0,00303812 | 0,00107843 | 2,81718218 | 0,00493284 | 0,33543289 |
| MMP-10 | OID00527 | 0,0036006 | 0,00143939 | 2,5014784 | 0,01251444 | 0,85098161 |
| ***Decreasing proteins as age advances*** | | | | | | |
| CD8A | OID05124 | -0,011782 | 0,00158033 | -7,4553996 | 1,8318E-13 | 1,2456E-11 |
| NT-3 | OID00554 | -0,0052771 | 0,00097991 | -5,3852957 | 8,8691E-08 | 6,031E-06 |
| TNFB | OID00561 | -0,0041194 | 0,00107143 | -3,844788 | 0,00012769 | 0,00868305 |
| ADA | OID00560 | -0,003303 | 0,00090344 | -3,6560407 | 0,00026845 | 0,01825467 |
| SIRT2 | OID00538 | -0,0065077 | 0,00213104 | -3,053754 | 0,00231522 | 0,15743466 |
| TRANCE | OID00521 | -0,0044385 | 0,00154933 | -2,8647908 | 0,00425353 | 0,28924034 |
| CD5 | OID00531 | -0,0020439 | 0,00074122 | -2,7574605 | 0,00592327 | 0,40278247 |
| OSM | OID00494 | -0,0053096 | 0,00193691 | -2,7412528 | 0,00622138 | 0,42305417 |
| CD244 | OID00477 | -0,0020199 | 0,00078872 | -2,5609743 | 0,01057263 | 0,71893899 |
| AXIN1 | OID00487 | -0,0063633 | 0,00251495 | -2,5301981 | 0,01154076 | 0,78477144 |

**eTable 7.** Association of inflammatory proteins with age changes in STOP (unadjusted).

| Protein | OlinkID | Estimate | Std. Error | t value | Pval | P_bonf* |  |
| --- | --- | --- | --- | --- | --- | --- | --- |
| ***Higher in females*** | | | | | | | |
| OPG | OID00479 | 0,16046115 | 0,02001874 | 8,01554675 | 2,8246E-15 | 1,9207E-13 |  |
| CCL28 | OID00539 | 0,16765803 | 0,0231159 | 7,25293196 | 7,7574E-13 | 5,275E-11 |  |
| CXCL5 | OID00520 | 0,44158128 | 0,06575897 | 6,71514846 | 3,0283E-11 | 2,0592E-09 |  |
| Flt3L | OID00533 | 0,1497537 | 0,02409382 | 6,21544141 | 7,2933E-10 | 4,9594E-08 |  |
| TNFB | OID00561 | 0,14909582 | 0,02572654 | 5,79540974 | 8,9322E-09 | 6,0739E-07 |  |
| FGF-23 | OID00507 | 0,13279904 | 0,02589838 | 5,12769755 | 3,4732E-07 | 2,3618E-05 |  |
| LIF-R | OID00511 | 0,08479509 | 0,01703595 | 4,97742152 | 7,4928E-07 | 5,0951E-05 |  |
| CXCL1 | OID00496 | 0,23493172 | 0,05016718 | 4,6829765 | 3,1863E-06 | 0,00021667 |  |
| IL-12B | OID00523 | 0,13772246 | 0,03371525 | 4,08487178 | 4,7354E-05 | 0,00322011 |  |
| CXCL11 | OID00486 | 0,16498904 | 0,04432212 | 3,72249891 | 0,00020742 | 0,01410442 |  |
| IFN-gamma | OID05547 | 0,20647895 | 0,0586558 | 3,52017964 | 0,0004492 | 0,03054551 |  |
| IL7 | OID00478 | 0,11707187 | 0,03994427 | 2,93087998 | 0,00345107 | 0,23467287 |  |
| CDCP1 | OID00476 | 0,08973924 | 0,03117356 | 2,87869763 | 0,0040718 | 0,27688235 |  |
| CXCL9 | OID00490 | 0,13283026 | 0,04710789 | 2,81970292 | 0,00489454 | 0,33282882 |  |
| CSF-1 | OID00562 | 0,03223334 | 0,01145241 | 2,81454543 | 0,00497318 | 0,33817656 |  |
| IL6 | OID00482 | 0,11095763 | 0,03989353 | 2,78134417 | 0,00550744 | 0,37450582 |  |
| ***Higher in males*** | | | | | | | |
| TRAIL | OID00488 | -0,1428299 | 0,01906012 | -7,4936538 | 1,3891E-13 | 9,4461E-12 |  |
| ADA | OID00560 | -0,1484157 | 0,02154531 | -6,8885385 | 9,546E-12 | 6,4913E-10 |  |
| TRANCE | OID00521 | -0,245968 | 0,03691127 | -6,6637635 | 4,2425E-11 | 2,8849E-09 |  |
| DNER | OID01213 | -0,0744598 | 0,01341242 | -5,5515514 | 3,561E-08 | 2,4215E-06 |  |
| MCP-1 | OID00484 | -0,1259034 | 0,02448988 | -5,141037 | 3,2409E-07 | 2,2038E-05 |  |
| IL18 | OID00501 | -0,1600322 | 0,03123477 | -5,1235263 | 3,5491E-07 | 2,4134E-05 |  |
| IL-15RA | OID00514 | -0,0809162 | 0,01584078 | -5,1080948 | 3,844E-07 | 2,6139E-05 |  |
| GDNF | OID00475 | -0,0976057 | 0,01977414 | -4,9360303 | 9,2271E-07 | 6,2744E-05 |  |
| 4E-BP1 | OID00536 | -0,2779028 | 0,05853098 | -4,7479612 | 2,3306E-06 | 0,00015848 |  |
| TWEAK | OID00555 | -0,0759367 | 0,01703968 | -4,456461 | 9,1992E-06 | 0,00062554 |  |
| STAMBP | OID00558 | -0,1403405 | 0,0357248 | -3,9283778 | 9,094E-05 | 0,00618391 |  |
| PD-L1 | OID00518 | -0,0856029 | 0,02239096 | -3,8231025 | 0,0001393 | 0,00947248 |  |
| SCF | OID00500 | -0,0759988 | 0,01992182 | -3,81485 | 0,00014398 | 0,00979031 |  |
| TNFRSF9 | OID00553 | -0,0787367 | 0,02089014 | -3,7690867 | 0,0001727 | 0,01174327 |  |
| IL-18R1 | OID00517 | -0,0871933 | 0,02417798 | -3,6063087 | 0,00032475 | 0,02208304 |  |
| CCL4 | OID00498 | -0,1079757 | 0,03485685 | -3,0976894 | 0,00200062 | 0,13604195 |  |
| EN-RAGE | OID00541 | -0,1082921 | 0,03696331 | -2,9297177 | 0,0034639 | 0,23554534 |  |
| IL10 | OID00528 | -0,0992259 | 0,03471201 | -2,8585473 | 0,0043375 | 0,29494971 |  |
| IL-10RB | OID00515 | -0,0424538 | 0,0161233 | -2,6330727 | 0,00858223 | 0,58359168 |  |
| CCL11 | OID00505 | -0,0700524 | 0,02676846 | -2,6169733 | 0,00899503 | 0,61166183 |  |
| AXIN1 | OID00487 | -0,1494215 | 0,06091375 | -2,4530006 | 0,01432396 | 0,97402936 |  |

**eTable 8.** Association of inflammatory proteins with each sex in STOP (unadjusted)

| Protein | OlinkID | | Estimate | | | Std. Error | | t value | | Pval | | | P_bonf* | | |  |  |
| --- | --- | --- | --- | --- | --- | --- | --- | --- | --- | --- | --- | --- | --- | --- | --- | --- | --- |
| ***Increasing protein with BMI*** | | | | | | | | | | | | | | | |  |  |
| HGF | | OID00522 | | 0,03129603 | | | 0,00301196 | | 10,3905834 | | | 7,6843E-24 | | | 5,2253E-22 | | |
| TNFSF14 | | OID00506 | | | 0,03746249 | | 0,003882 | | 9,65031117 | | 6,0664E-21 | | | 4,1252E-19 | | |  |
| IL-18R1 | | OID00517 | | | 0,02944324 | | 0,00355377 | | 8,2850766 | | 4,799E-16 | | | 3,2633E-14 | | |  |
| VEGFA | | OID00472 | | | 0,02109803 | | 0,00256752 | | 8,21729647 | | 8,102E-16 | | | 5,5094E-14 | | |  |
| IL6 | | OID00482 | | | 0,03875751 | | 0,0058463 | | 6,62940792 | | 6,1118E-11 | | | 4,156E-09 | | |  |
| OSM | | OID00494 | | | 0,0433316 | | 0,00683308 | | 6,34144675 | | 3,7594E-10 | | | 2,5564E-08 | | |  |
| CCL3 | | OID00532 | | | 0,02676738 | | 0,0044828 | | 5,97112972 | | 3,5086E-09 | | | 2,3859E-07 | | |  |
| TRANCE | | OID00521 | | | 0,03273064 | | 0,00560903 | | 5,8353455 | | 7,729E-09 | | | 5,2557E-07 | | |  |
| CDCP1 | | OID00476 | | | 0,02587911 | | 0,00457302 | | 5,65908836 | | 2,1043E-08 | | | 1,4309E-06 | | |  |
| FGF-21 | | OID00512 | | | 0,05781261 | | 0,01081204 | | 5,34705935 | | 1,1603E-07 | | | 7,89E-06 | | |  |
| TRAIL | | OID00488 | | | 0,01486306 | | 0,00294637 | | 5,04452698 | | 5,6018E-07 | | | 3,8092E-05 | | |  |
| IL18 | | OID00501 | | | 0,02309854 | | 0,00460901 | | 5,01160958 | | 6,6164E-07 | | | 4,4991E-05 | | |  |
| CSF-1 | | OID00562 | | | 0,00828279 | | 0,0017364 | | 4,77009019 | | 2,1797E-06 | | | 0,00014822 | | |  |
| IL-10RB | | OID00515 | | | 0,01094018 | | 0,00239739 | | 4,56336701 | | 5,805E-06 | | | 0,00039474 | | |  |
| MCP-1 | | OID00484 | | | 0,01667399 | | 0,00381605 | | 4,36943192 | | 1,4058E-05 | | | 0,00095591 | | |  |
| CCL19 | | OID00513 | | | 0,0288663 | | 0,00683952 | | 4,22051601 | | 2,7101E-05 | | | 0,00184284 | | |  |
| GDNF | | OID00475 | | | 0,01185371 | | 0,00301247 | | 3,93487685 | | 9,0302E-05 | | | 0,0061405 | | |  |
| SIRT2 | | OID00538 | | | 0,03083273 | | 0,00786295 | | 3,92126541 | | 9,5458E-05 | | | 0,00649114 | | |  |
| CD6 | | OID00499 | | | 0,01543264 | | 0,00401959 | | 3,83935477 | | 0,00013287 | | | 0,00903503 | | |  |
| CCL4 | | OID00498 | | | 0,01971197 | | 0,00518177 | | 3,80410027 | | 0,00015291 | | | 0,01039764 | | |  |
| IL-12B | | OID00523 | | | 0,01860742 | | 0,00513296 | | 3,62508598 | | 0,00030671 | | | 0,02085603 | | |  |
| STAMBP | | OID00558 | | | 0,01977302 | | 0,00546685 | | 3,61689214 | | 0,00031642 | | | 0,02151639 | | |  |
| LAP TGF-β1 | | OID00480 | | | 0,01304858 | | 0,00370641 | | 3,5205437 | | 0,00045445 | | | 0,03090229 | | |  |
| FGF-23 | | OID00507 | | | 0,01415012 | | 0,00406579 | | 3,48028574 | | 0,00052735 | | | 0,03586008 | | |  |
| CD5 | | OID00531 | | | 0,00903921 | | 0,0026698 | | 3,38573012 | | 0,00074369 | | | 0,05057108 | | |  |
| CCL20 | | OID00556 | | | 0,02284224 | | 0,00704474 | | 3,24245194 | | 0,00123301 | | | 0,08384454 | | |  |
| IL10 | | OID00528 | | | 0,01550928 | | 0,0051923 | | 2,98697965 | | 0,00290151 | | | 0,19730274 | | |  |
| CD40 | | OID00542 | | | 0,00823126 | | 0,00283657 | | 2,90183115 | | 0,00380937 | | | 0,25903702 | | |  |
| IL-15RA | | OID00514 | | | 0,00639743 | | 0,00234934 | | 2,72307156 | | 0,00660541 | | | 0,44916776 | | |  |
| CD244 | | OID00477 | | | 0,00772904 | | 0,00283896 | | 2,7224849 | | 0,00661704 | | | 0,44995873 | | |  |
| AXIN1 | | OID00487 | | | 0,02487218 | | 0,00917665 | | 2,71037709 | | 0,00686126 | | | 0,46656597 | | |  |
| TGF-α | | OID00503 | | | 0,00688773 | | 0,00265811 | | 2,59121031 | | 0,00973411 | | | 0,66191946 | | |  |
| ***Decreasing protein with BMI*** | | | | | | | | | | | | | | | |  |  |
| NT-3 | | OID00554 | | | -0,0169193 | | 0,00342807 | | -4,9355114 | | 9,6866E-07 | | | -0,0169193 | | |  |
| SCF | | OID00500 | | | -0,010338 | | 0,0029911 | | -3,4562453 | | 0,00057596 | | | -0,010338 | | |  |
| CCL28 | | OID00539 | | | -0,011435 | | 0,00362474 | | -3,1547025 | | 0,00166529 | | | -0,011435 | | |  |

**eTable 9.** Association of inflammatory proteins with BMI in STOP (unadjusted)

| **Protein** | **OlinkID** | **Sum Sq** | | **Mean Sq** | | **F value** | | **Pval** | | **P_bonf** | |  |
| --- | --- | --- | --- | --- | --- | --- | --- | --- | --- | --- | --- | --- |
| CDCP1 | OID00476 | 9,05179228 | | 4,52589614 | | 18,0353544 | | 2,1614E-08 | | 1,4697E-06 | |  |
| IL8 | OID00471 | 8,37957435 | | 4,18978718 | | 14,7195287 | | 5,2416E-07 | | 3,5643E-05 | |  |
| Flt3L | OID00533 | 4,18709531 | | 2,09354766 | | 13,5585087 | | 1,6102E-06 | | 0,00010949 | |  |
| VEGFA | OID00472 | 1,96340519 | | 0,98170259 | | 11,7613329 | | 9,2047E-06 | | 0,00062592 | |  |
| HGF | OID00522 | 2,29399526 | | 1,14699763 | | 9,49962516 | | 8,3452E-05 | | 0,00567471 | |  |
| CCL11 | OID00505 | 3,41598626 | | 1,70799313 | | 8,38969125 | | 0,00024728 | | 0,01681474 | |  |
| FGF-21 | OID00512 | 22,5315697 | | 11,2657849 | | 7,85172404 | | 0,00041906 | | 0,02849632 | |  |
| MCP-1 | OID00484 | 2,72942002 | | 1,36471001 | | 7,7408616 | | 0,00046723 | | 0,03177147 | |  |
| IL18 | OID00501 | 3,50715759 | | 1,7535788 | | 6,76281619 | | 0,00122154 | | 0,0830644 | |  |
| IL-18R1 | OID00517 | 1,89627432 | | 0,94813716 | | 5,83687134 | | 0,00304056 | | 0,20675802 | |  |
| CCL28 | OID00539 | 1,8212031 | | 0,91060155 | | 5,76952381 | | 0,00324933 | | 0,22095448 | |  |
| CCL25 | OID00551 | 3,10419607 | | 1,55209804 | | 5,69125078 | | 0,0035101 | | 0,23868647 | |  |
| NT-3 | OID00554 | 1,51794123 | | 0,75897062 | | 5,26405466 | | 0,00535059 | | 0,36384023 | |  |
| MMP-1 | OID00510 | 10,6485804 | | 5,3242902 | | 4,8632118 | | 0,0079499 | | 0,54059349 | |  |
| SCF | OID00500 | 1,04805696 | | 0,52402848 | | 4,84024113 | | 0,00813245 | | 0,55300643 | |  |
|  |  |  |  | |  | |  | |  | |  | |

**eTable 10.** Association of inflammatory proteins with smoking status in STOP (unadjusted)

| Protein | OlinkID | Sum Sq | Mean Sq | F value | Pval | P_bonf* |
| --- | --- | --- | --- | --- | --- | --- |
| AXIN1 | OID00487 | 194,557513 | 97,2787566 | 117,988116 | 4,4762E-47 | 3,0438E-45 |
| SIRT2 | OID00538 | 115,294907 | 57,6474533 | 93,5105198 | 3,4909E-38 | 2,3738E-36 |
| STAMBP | OID00558 | 50,556728 | 25,278364 | 83,7821114 | 1,4883E-34 | 1,0121E-32 |
| CD40 | OID00542 | 5,31570741 | 2,65785371 | 28,9622242 | 5,5884E-13 | 3,8001E-11 |
| TNFSF14 | OID00506 | 9,03852455 | 4,51926227 | 23,2148686 | 1,3441E-10 | 9,1396E-09 |
| ADA | OID00560 | 4,43425704 | 2,21712852 | 17,548013 | 3,163E-08 | 2,1508E-06 |
| LAP TGFβ1 | OID00480 | 5,68790233 | 2,84395116 | 17,3955016 | 3,6665E-08 | 2,4932E-06 |
| CD5 | OID00531 | 2,82420084 | 1,41210042 | 16,6650394 | 7,4439E-08 | 5,0618E-06 |
| CD6 | OID00499 | 6,18736261 | 3,09368131 | 15,9998129 | 1,4198E-07 | 9,6547E-06 |
| CXCL5 | OID00520 | 34,2243491 | 17,1121746 | 14,4896868 | 6,1671E-07 | 4,1936E-05 |
| CXCL1 | OID00496 | 18,8646151 | 9,43230756 | 13,9983213 | 9,954E-07 | 6,7687E-05 |
| IL7 | OID00478 | 8,68936352 | 4,34468176 | 10,2249446 | 3,9888E-05 | 0,00271239 |
| CXCL6 | OID00534 | 7,17215264 | 3,58607632 | 9,30897267 | 9,8081E-05 | 0,0066695 |
| CD244 | OID00477 | 1,42315966 | 0,71157983 | 7,2990289 | 0,00071004 | 0,04828296 |
| MMP-1 | OID00510 | 12,7555511 | 6,37777553 | 6,11099104 | 0,00229573 | 0,15610982 |
| 4E-BP1 | OID00536 | 9,13278501 | 4,5663925 | 4,89404661 | 0,00765786 | 0,5207346 |
| CCL11 | OID00505 | 1,80872252 | 0,90436126 | 4,69917425 | 0,00928956 | 0,63168996 |
| VEGFA | OID00472 | 0,77537551 | 0,38768776 | 4,65464784 | 0,00970883 | 0,66020021 |
| CXCL9 | OID00490 | 5,2965428 | 2,6482714 | 4,43662831 | 0,01205207 | 0,81954098 |
| TWEAK | OID00555 | 0,69611269 | 0,34805635 | 4,40821248 | 0,0123966 | 0,84296852 |

**eTable 11.** Association of inflammatory proteins with sample lab in STOP (unadjusted).
